# Sodoku

**Published:** 1917-09

**Authors:** 


**Sodoku.** S. Costa and J. Triosiere, Soc. Med. des Hop., Nov. 24, 1916. This is a fever caused by the bite of the ordinary rat, the incubation being 2 or 3 days, the febrile stage 10-27 days. Miyake has described a spirochete visible with the ultra-microscope. It is rare in Europe. The present case is described as a septicaemia and it seems doubtful whether it is the specific infection with which Miyake has connected a spirochete. Sepsis due to bites of various animals is not especially uncommon.

